# Development and Evaluation of Crocetin-Functionalized Pegylated Magnetite Nanoparticles for Hepatocellular Carcinoma

**DOI:** 10.3390/molecules28072882

**Published:** 2023-03-23

**Authors:** Sulafa Ibrahim, Badriya Baig, Soleiman Hisaindee, Hussein Darwish, Ashraf Abdel-Ghany, Hesham El-Maghraby, Amr Amin, Yaser Greish

**Affiliations:** 1 Department of Chemistry, United Arab Emirates University, Al Ain P.O. Box 15551, United Arab Emirates; sulafa.s.abdelhalim@gmail.com (S.I.); soleiman.hisaindee@uaeu.ac.ae (S.H.); habdelrehim@uaeu.ac.ae (H.E.-M.); 2 Department of Biology, United Arab Emirates University, Al Ain P.O. Box 15551, United Arab Emirates; 200918733@uaeu.ac.ae; 3 Department of Glass Research, National Research Centre, Dokki, Cairo 12622, Egypt; husseinnrc@yahoo.com; 4 Department of Inorganic Chemistry, National Research Centre, Dokki, Cairo 12622, Egypt; achraf_28@yahoo.com; 5 Department of Ceramics, National Research Centre, Dokki, Cairo 12622, Egypt; 6 Zayed Centre for Health Sciences, United Arab Emirates University, Al Ain P.O. Box 15551, United Arab Emirates

**Keywords:** magnetite nanoparticles, PEG, crocetin, hepatocellular carcinoma

## Abstract

Liver cancer remains among the leading causes of cancer-related deaths worldwide. This is due to many reasons, including limitations of available drugs, late diagnosis due to the overlapping symptoms with many other liver diseases, and lack of effective screening modalities. Compared to conventional chemotherapy, targeted drug delivery systems are advantageous in many ways, as they minimize drug resistance and improve therapeutic value for cancer patients. Nanomaterials, in general, and nanoparticles, in particular, possess nm size, which provides a high surface area for a great extent of functionalization to be used for the targeted delivery of cancer drugs. Amongst the different formulations of nanoparticles, magnetic nanoparticles (MNPs) have unique chemical and physical characteristics and magnetic behavior, making them preferable candidates as a core for drug delivery systems. To maintain the nanosized structure of MNPs, a polymeric coating is usually applied to maintain the nanoparticles dispersed in the solution. Moreover, the polymeric coating provides a plate form for carrying drug molecules on its surface. In the present study, poly(ethylene glycol) (PEG)-coated MNPs were successfully synthesized, where the optimum concentration of PEG on the surface of the MNPs was investigated. The PEG-coated MNPs were further coated with crocetin at different concentrations. The crocetin-coated pegylated MNPs were evaluated in vitro using a hepatic cell line (HepG2) for up to 72 h. Results showed good release kinetics under acidic and neutral conditions. The optimally prepared drug delivery system showed a high potential for reducing the HepG2 cell proliferation in vitro using an MTT assay. The calculated IC50 for Cro-PEG-MNPs were 0.1019, 0.0903, and 0.0462 mg/mL of 5×, 10× and 20×, respectively.

## 1. Introduction

Magnetite nanoparticles (MNPs, Fe_3_O_4_) are a type of iron oxide that have received a great deal of focus due to their multifunctional properties, such as small size, hence high surface area, superparamagnetism, low toxicity, good biodegradability, good colloidal stability, and relatively easy, rapid, and inexpensive production [1,2,3]. Especially in biomedical applications, MNPs have been extensively utilized for different applications, including molecular imaging, hyperthermia, cell/protein separation, drug/gene delivery, tissue repair, and stem cell tracking [3]. Iron oxides exist in three main compositions: maghemite (γ-Fe_2_O_3_), hematite (α-Fe_2_O_3_), and magnetite (Fe_3_O_4_) [4]. These iron oxides not only differ in their composition and crystal structure, but they also exhibit variable magnetic properties [5]. Magnetite exhibits the most potent magnetism among all the transition metal oxides [6]. MNPs can be synthesized by different methods, including physical, chemical, and biological methods. The choice of synthesis method helps produce MNPs of the desired shape, size, structure, colloidal stability, and magnetic properties [4]. Among all the synthesis methods, chemical methods are the most utilized due to their relative simplicity and high yield. Among the different chemical methods, co-precipitation is the most exploited method. In this method, MNPs are prepared from aqueous salt solutions by the addition of a base under an inert atmosphere at low temperatures [7]. The reaction is simply presented below in Equation (1):

Fe^2+^ + 2Fe^3+^ + 8OH^−^ → Fe_3_O_4_ + 4H_2_O (1)

Several factors dictate the size and morphology of the formed MNPs. These factors include the type of salts used, such as chlorides, sulfates, nitrates, perchlorates, etc., the ratio of ferric (Fe^3+^) to ferrous (Fe^2+^) ions, the pH value, the reaction temperature, the ionic strength of the media, and the other reaction parameters (such as stirring rate and feeding rate of the fundamental solution). The pH between 8 and 14 is the expected range for complete precipitation with a stoichiometric ratio of 2/1 (Fe^3+^/F^2+^) [1]. Sun and his coworkers prepared supermagnetic iron oxide nanoparticles capped with oleic acid by mixing 1:1.75 ferrous to ferric ions at 80 °C and under inert conditions. Using ammonia solution, the pH of the solution was brought up to above 10. As a result, the MNPs had a size of 12 nm, and the oleic-acid-coated MNPs had a hydrodynamic size of around 100 nm [8]. In another study, MNPs were prepared by dropwise addition of sodium hydroxide to a mixture of 1:2 ferrous to ferric iron ions under vigorous stirring at 2000 rpm. The obtained MNPs had an average size of 11 nm and exhibited supermagnetization [9].

MNPs are usually crystalline. Those nanoparticles only show a magnetic behavior in the presence of an external magnetic field and return to the unmagnetized condition when the external magnetic field is removed. This phenomenon is called superparamagnetism; hence MNPs are also known as superparamagnetic iron oxide nanoparticles (SPIONs). This characteristic is what makes MNPs promising to be used in biomedical applications. MNPs can be guided by an external magnetic field to be absorbed into an organ, tissue, or tumor for targeted therapy or can be heated by an alternating magnetic field for use in hyperthermia. MNPs are also used as a contrast agent in magnetic resonance imaging (MRI) [1].

One of the challenges of dealing with MNPs is their spontaneous agglomeration; hence an overall particle size is higher than that of individual MNPs. These large agglomerates have their net polarization/dipole without an external magnetic field, which may lead to a loss of the superparamagnetic properties of MNPs. The nanosized dimensions also increase the chemical reactivity of these particles, resulting in a faster degradation in biological systems, hence a lack of stability of the bare MNPs, which tends to restrict its application in biomedical applications [7,10]. Therefore, surface modification of the bare MNPs is critical to achieving colloidal stability. Colloidal stability refers to the ability to resist clumping/agglomeration and remain monodispersed in a suspension/solution as a colloid [4]. To prevent the aggregation of MNPs, the surface of the nanoparticles must be modified with biodegradable and biocompatible polymers, ligands, or surfactants [11,12]. The coating gives many advantages, such as an increase in the colloidal stability of the MNPs system; a reduction in the size of MNPs as a result of lowering the agglomeration; a reduction in the toxicity of MNPs, offering binding sites for further functionalization with a therapeutic molecule or/and targeting ligand; and control of the surface charge and solubility [10,13,14]. Polyethylene Glycol (PEG) is a long linear or branched polymeric chain of polyether terminated with hydroxyl groups. It is widely used for biomedical applications as a surface coating material for NPs due to its biocompatibility and ability to reduce nonspecific protein adsorption and clearance by RES macrophages [15]. This is because a dense layer of PEG gives hydrophilicity to the surface of MNPs, which offers the NPs better water dispersity and steric stability than bare MNPs. In addition, the chemical functionality along the PEG coating would enhance the potential for the conjugation of other targeting ligands [13,16].

Furthermore, using PEG during the synthesis of MNPs provides stability between the particles via steric repulsion [17]. This prevents the unnecessary accumulation observed with bare MNPs. Subsequently, smaller particles size of MNPs with better dispersion will be obtained. A research study was carried out to construct PEG-coated MNPs as contrast agents for long-lasting real-time monitoring of tumor evolution [18]. The stability of the MNPs system depended on optimizing the suitable molecular weight of PEG and studying the biocompatibility of the fabricated PEG-MNPs in vivo and in vitro [18].

A multifunctional delivery system can be designed by Incorporating different functioning molecules onto the surface of the polymeric coating. A growing interest in the use of natural products for the treatment of cancer has been observed. This is attributed to the therapeutic effects of natural herbs because of their low or no toxicity [19]. *Crocus sativus* L. (*C. sativus*), commonly known as saffron, has four main biologically active components that were shown to have anticancer [20], anti-microbial [21], antimutagenic [22], and anti-genotoxic properties [23]. Those biologically active components are crocin, crocetin, picrocrocin (responsible for the taste), and Safranal (responsible for the aroma of saffron). Crocin is a mono-glycosyl or di-glycosyl polyene ester, while crocetin is the dicarboxylic acid precursor of crocin. Both crocin and crocetin are carotenoids and are responsible for the pigment of saffron [24]. Although crocin and crocetin are structurally related, the difference in the terminal groups makes crocin more soluble than crocetin.

Moreover, in a study comparing the cytotoxic effect of crocin and crocetin on five human cancer lines (A549, HepG2, HCT-116, SK-OV-3, and HeLa cells), crocetin had a 5- to 18-fold higher cytotoxicity than crocin. It was attributed to the enhanced levels of production of reactive oxygen species (ROS) produced by crocetin compared to crocin. This indicates that the two compounds have different mechanisms for their cytotoxic effect [25]. Another study looked at crocetin’s anticancer effect on human esophageal squamous cell carcinoma KYSE-150 cells. They found that crocetin affected cell proliferation and migration, and induced apoptosis of the cell line [26,27].

This study aims to develop an optimized system of crocetin-coated PEG-functionalized MNPs, and the evaluation of their efficiency toward hepatic cancer cells in vitro. Optimizing the process of coating MNPs with PEG at different concentrations and the subsequent crocetin functionalization to varying concentrations of crocetin will be outlined. The delivery system will then be studied for its release kinetics and in vitro effect on a model HepG2 liver cancer cell line. Given the potential of crocetin as an anticancer drug, this work provides a drug delivery composite system that has not been studied before for the treatment of hepatic carcinoma. Moreover, it provides a higher potential for cancer treatment than crocin-coated magnetite nanoparticles that were also prepared and evaluated in our laboratories.

## 2. Results and Discussion

### 2.1. Characterization of Pristine Magnetite NPs

Magnetite (Fe_3_O_4_) is a combined structure of FeO and Fe_2_O_3_ within a unified crystal lattice. Most of the published research following the co-precipitation method to prepare MNPs recommends an inert atmosphere to avoid the formation of phase-impure MNPs. However, if the reaction media were pre-adjusted to be highly alkaline before adding Fe^2+^ and Fe^3+^ ions, then MNPs would be spontaneously precipitated as the highest thermodynamically stable phase [28]. Therefore, even though all preparations were carried out in the presence of air, phase-pure Fe_3_O_4_ will be the end product. In the current study, aqueous media used to prepare MNPs were pre-adjusted to a pH 13 using NaOH to avoid the formation of other non-magnetite iron oxides. To determine the phase purity of the precipitated MNPs, XRD, FT-IR, and TGA analyses were conducted.

**Figure 1:**
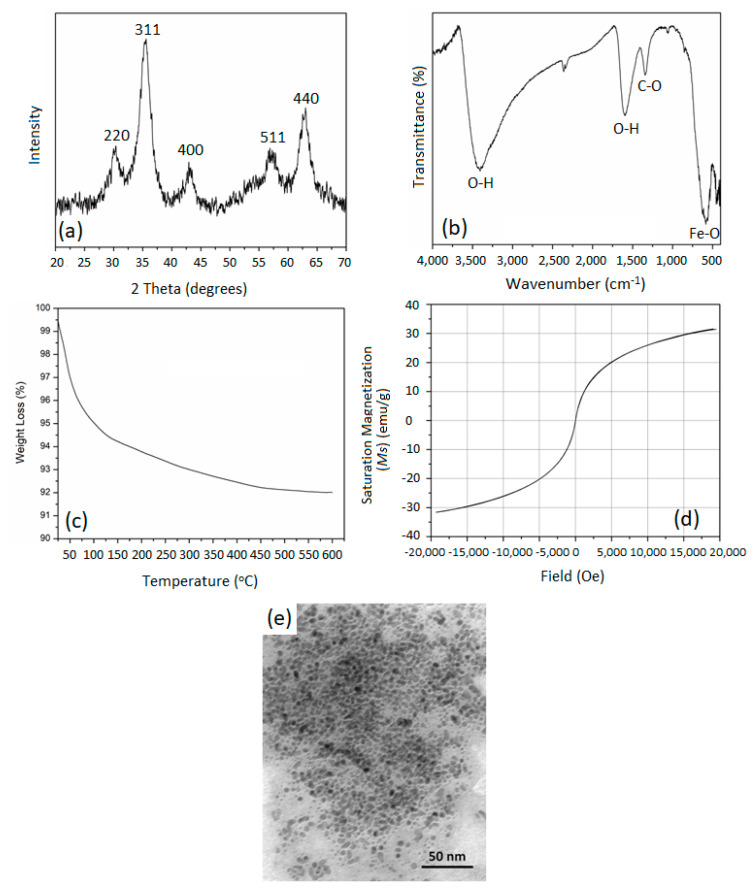
(**a**) XRD pattern, (**b**) FT-IR spectrum, (**c**) TGA thermogram, (**d**) magnetization thermogram, and (**e**) TEM micrograph of the as-prepared magnetite nanoparticles.

The XRD pattern in Figure 1a shows the phase purity and crystallinity of the as-prepared MNPs. The peaks formed to align with the JCPDS (00-019-0629) pattern of the pure magnetite phase, including the characteristic peaks at 2θ values 29.9° (220), 35.2° (311), 43.1° (400), 57.2° (511), and 62.8° (440) [29]. The relative broadness of the XRD peaks indicates that the as-prepared MNPs are in the nanoscale [16]. The absence of hematite or other hydroxides in the XRD patterns of the MNPs prepared in the current study confirms the phase purity of the prepared MNPs. However, possible surface oxidation of the precipitated MNPs may result in forming a thin layer of maghemite (γ-Fe_2_O_3_), where all Fe^2+^ ions are oxidized to Fe^3+^ ions. Both the magnetite and maghemite phases share the same crystal structure in the XRD pattern, hence they cannot be distinguished from each other upon the XRD analysis.

The FTIR spectrum in Figure 1b for the as-prepared MNPs demonstrated bands at wavenumbers of 443.6, 581.2, and 623.7 cm^−1^ that are characteristic of the Fe-O stretching mode of absorption of the magnetite phase, while the broad band around 3400 cm^−1^ and the band at 1596.9 cm^−1^ are characteristic of the stretching and bending modes of absorption of the O-H bond, respectively. This is attributed to the presence of physically adsorbed water molecules. An additional band around 1342.2 cm^−1^ was observed, and is related to physically adsorbed CO_2_ molecules, since the preparation of MNPs was carried out in air. This has been further confirmed in the TGA thermogram of the as-prepared MNPs shown in Figure 1c. The TGA thermogram showed weight loss values of about 6% taking place at around 100 °C, which is attributed to the evaporation of the physically adsorbed water. This loss continued to occur more slowly as the temperature increased. This could be attributed to the presence of multilayers of water of hydration on the surfaces of the MNPs. Furthermore, we expect high agglomeration between the magnetite NPs, which could result in water molecules getting trapped between the MNPs and later lost in TGA at temperatures higher than 100 °C.

The magnetic hysteresis of pure MNP is illustrated in Figure 1d. The magnetization measurements of pure MNPs as a function of an applied field at room temperature showed a maximum magnetization of 31.6 emu/g, which is lower than what is reported in the literature [30]. This could be attributed to the high agglomeration of the MNPs, which lowers the surface area and hence gives lower magnetization. This could also be attributed to the possible formation of an oxidized layer on the surfaces of the as-prepared MNPs. Moreover, the absence of coercivity in the magnetization hysteresis of the as-prepared MNPs indicates its superparamagnetic nature. Considering the characterization of the as-prepared MNPs, no signs of non-magnetite phases were found. These findings further support their subsequent application as a drug delivery vehicle for cancer treatment. Figure 1e shows the TEM micrograph of the as-prepared MNPs, with a high extent of agglomeration. The micrograph indicates the formation of rounded NPs with homogeneous size distribution. The average sizes of the individual NPs were 4.4 ± 5 nm.

### 2.2. Characterization of PEG-Coated MNPs

MNPs have a large surface-to-volume ratio and therefore possess high surface energies. Consequently, they tend to aggregate to minimize these energies. Moreover, the bare MNPs have high chemical activity and are easily oxidized in the air, generally resulting in loss of magnetism and dispersibility [31]. Furthermore, in biomedical applications, neat MNPs undergo non-specific interactions with the serum proteins in vivo, leading to their rapid elimination from the body [29]. To address these limitations, the surfaces of the MNPs need to be hydrophilic for the NPs to be stable and have prolonged circulation inside the body. Hence, PEG was used as a coating for the MNPs. For the preparation of pegylated MNPs, we followed an ex situ approach via the direct immersion method. We treated the freshly prepared MNPs in solutions with PEG at different weight percentages, up to 18.5%. MNPs immersed in PEG were left to stir overnight to ensure a full coating of the MNPs. The next day, excess PEG was washed out by cycles of washing and decantation with deionized water. The formed PEG-MNPs were separated by decantation, centrifuged, washed, and then dried before being characterized for their composition.

**Figure 2:**
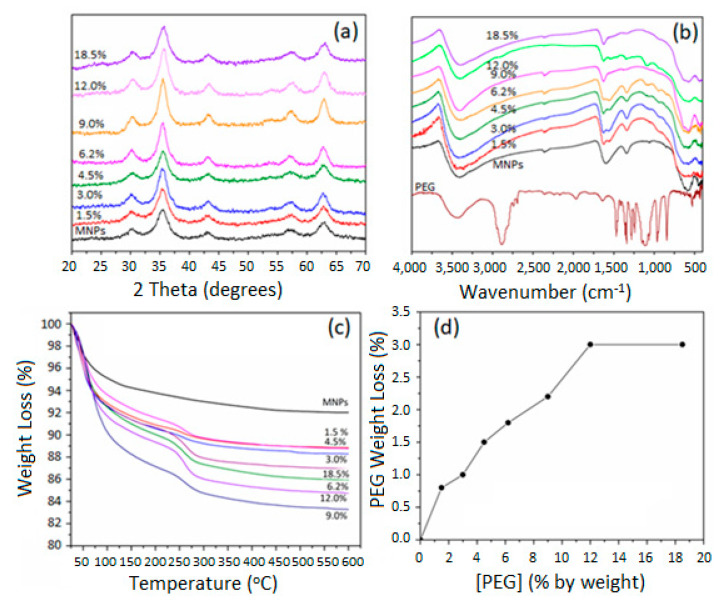
(**a**) XRD patterns of the PEG-coated MNPs as a function of the concentration of PEG, (**b**) FT-IR spectra of the PEG-coated MNPs as a function of the concentration of PEG, (**c**) TGA thermograms of the PEG-coated MNPs as a function of the concentration of PEG, (**d**) the extent of coating of MNPs with PEG as depicted from their TGA thermograms.

Figure 2a shows the XRD patterns of all PEG-coated MNPs as a function of the percentage of PEG used during the preparation. Compared with the XRD pattern of pure MNPs, all XRD patterns of the PEG-coated MNPs showed a consistent presence of pure MNPs with no signs of other non-magnetite phases. We observe that the peaks in all concentrations are well defined, indicating that the phase purity and crystallinity of the MNPs were not affected by the PEG coating on their surfaces. Moreover, the XRD peaks’ continued broadness suggests the presence of the PEG-coated MNPs in the form of nanoscale particulates [16]. Figure 2b shows the FTIR spectra of PEG-coated MNPs as a function of the [PEG] and they are compared with the FTIR spectrum of the as-prepared pure MNPs phase. Moreover, a spectrum of pure PEG is also shown in the respective spectra for comparison. In all spectra of pegylated MNPs, magnetite is represented by its bands at 430, 584, and 622 cm^−1^, with band intensities decreasing and shifting to lower wavenumbers as [PEG] was increased. The FTIR for the PEG-coated MNPs shows a shoulder absorption band at 951 cm^−1^ corresponding to -CH out-of-plane bending vibration of the PEG coating. Furthermore, the presence of PEG as a coating on the surfaces of MNPs is confirmed with the appearance of a new peak at 1100 cm^−1^, which is a signature peak of the C-O-C bond of PEG. The IR band at 2860 cm^−1^ corresponds to -C-H asymmetric stretching vibration, especially in the spectra of samples containing ≥4.6% of PEG. Moreover, the signature peaks at 3402 cm^−1^ due to hydroxyl stretching overlap with water and OH stretching vibrations in all samples, which could also be attributed to the physically adsorbed water molecules. An additional band was observed at 1596.9 cm^−1^ in the spectra of all samples. This is related to the bending mode of the O-H group of the physically attached water molecules. The intensity of this band was observed to decrease with increasing the [PEG] in the coating and could be attributed to the decrease in the extent of adsorption of water due to the presence of PEG as a coating. Taken together, these findings confirm the presence of PEG as a coating on the surfaces of MNPs [26].

**Scheme 1:**
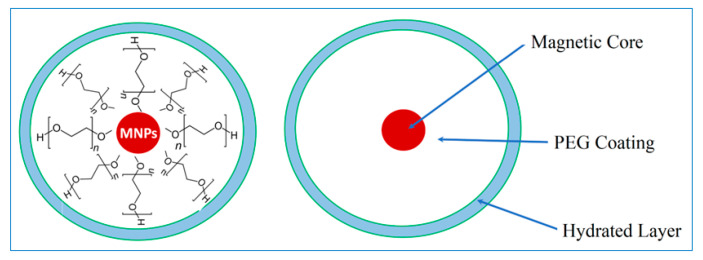
Mechanism of the formation of PEG and hydrated layers onto MNPs surfaces.

Pure MNPs showed an average weight loss of 6%, as was previously shown in Figure 1c. This was attributed to the removal of physically adsorbed water molecules. Figure 2c shows the TGA thermograms of PEG-coated MNPs as the function of the proportion of the [PEG]. Pure PEG showed an overall 100% weight loss at around 250 °C, which is attributed to the thermal and oxidative decomposition as well as the combustion of the PEG chains. On the other hand, TGA thermograms of PEG-coated MNPs indicated the presence of two stages of weight loss. The first event of weight loss accounts for the evaporation of physically and chemically adsorbed water, around 100 °C. The second weight loss event was observed around 250 ± 10 °C and is attributed to the degradation of PEG linked onto the surfaces of the MNPs. With the increasing percentage of PEG, we observe an increase in weight loss of around 100 °C attributed to the physically and chemically adsorbed water on the surface of PEG. This weight loss is then slowly stretched until the second major thermal event that takes place around 250 °C, corresponding to the degradation and thermal decomposition of the PEG coating. This stretch could be due to the increase in the thickness of the PEG coating layer as a result of the development of intermolecular forces, such as H-bonding and dispersion forces, amongst PEG molecules. Consequently, we observe, with the higher percentage of PEG, a more significant weight loss of around 250 °C, which indicates a multilayer formation of PEG on top of MNPs. A schematic representation of the PEG coating onto the surfaces of MNPs and the formation of a hydrated layer is represented in Scheme 1. Based on these discussions, Figure 2d shows a summary of the TGA weight loss as a function of the percentage of PEG added to MNPs. A maximum loading capacity was observed with 12% PEG-MNP, which was considered an optimum proportion for the further immobilization of crocetin molecules.

**Figure 3:**
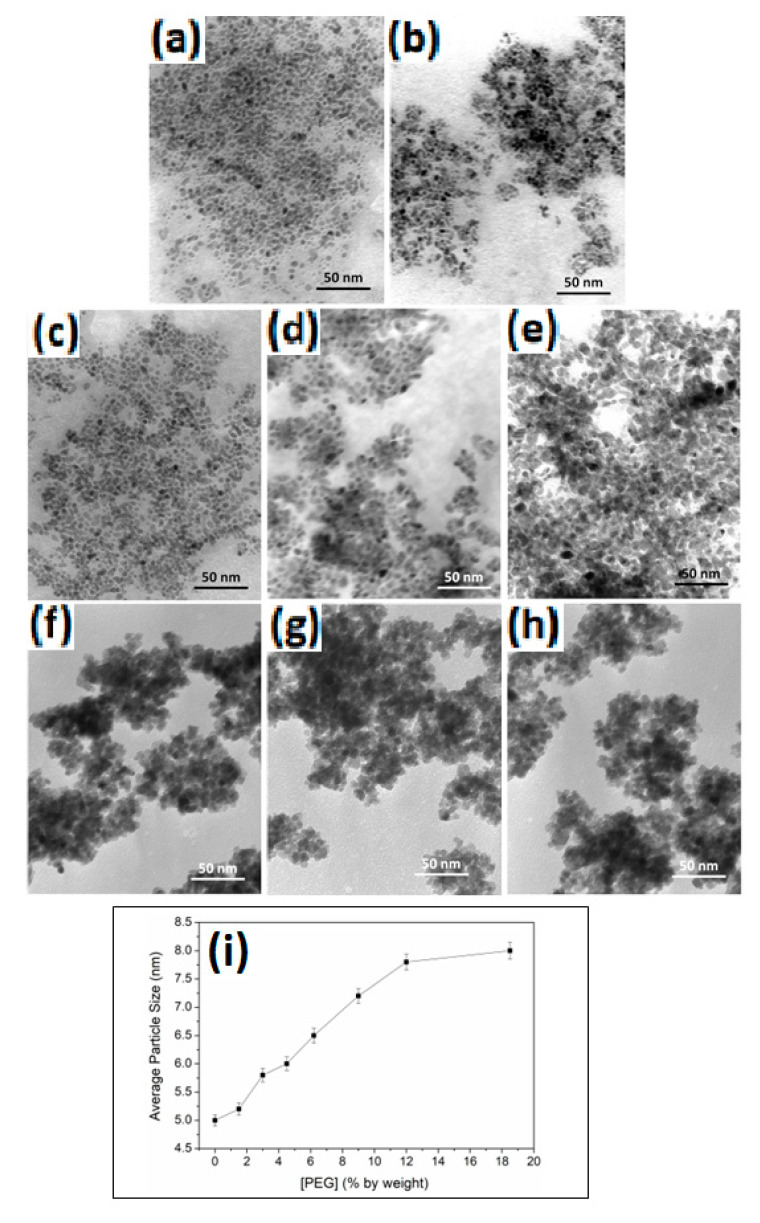
TEM micrographs of PEG-coated MNPs as a function of [PEG]: (**a**) 0%, (**b**) 1.5%, (**c**) 3.0%, (**d**) 4.5%, (**e**) 6.2%, (**f**) 9.0%, (**g**) 12.0%, and (**h**) 18.5% by weight, and (**i**) average particle size distribution as depicted from the TEM micrographs.

Figure 3 shows the TEM imaging of PEG-coated MNPs at different percentages of PEG. Overall, we observe that the presence of the coating reduced the extent of agglomeration compared to pure magnetite (Figure 3a). The coated MNPs retained the round shape with a homogeneous size distribution. The size of the particles increased slightly with the increasing PEG thickness. Compared to pure MNPs with a size 5.0 ± 0.12 nm, 1.5% PEG-MNPs had a size of 5.2 ± 0.11 nm, while 9% had a size of 7.2 ± 0.13 nm, and finally, 18.5% size was around 8.0 ± 0.15 nm. The trend of a systematic increase in the average particle size of the PEG-MNPs is consistent with the results shown in Figure 2d and confirms the extent of coating of the MNPs with PEG.

**Figure 4:**
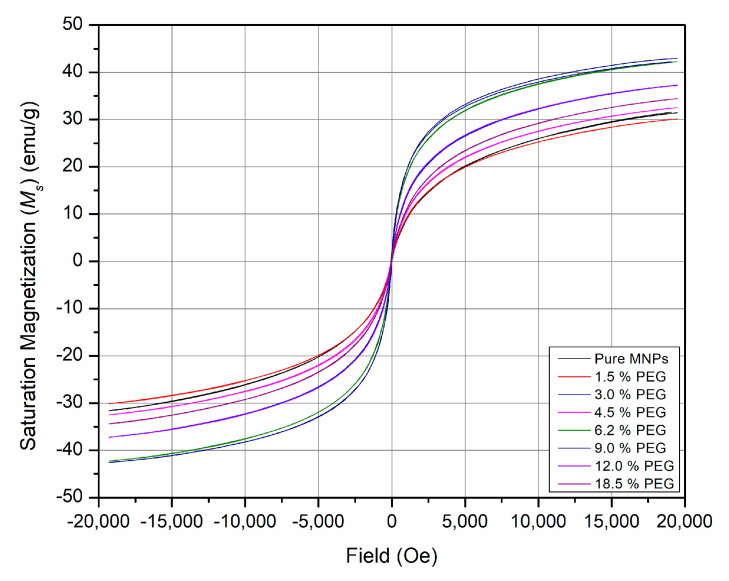
Magnetization hysteresis of the as-prepared PEG-coated MNPs as a function of the concentration of PEG.

The presence of a non-magnetic layer on a magnetic MNP’s core has always been believed to result in a decrease in the magnetic properties of the coated MNPs, hence decreasing their potential for biomedical applications [16,32,33,34]. In the current study, however, the magnetization results shown in Figure 4 for the PEG-coated MNPs as a function of the [PEG] indicate an erratic behavior. A saturation magnetization of 31 emu/g was observed for the as-prepared neat MNPs; Figure 1d. The presence of PEG as a coating on the surfaces of MNPs resulted in a general increase in the magnetization values compared to pure MNPs, especially at higher proportions of PEG. An increasing pattern was observed, where the presence of PEG in the coating increased the magnetization of the PEG-coated MNPs. For example, magnetization saturation values were 42.7, 37.2, and 34.4 for PEG-coated MNPs containing 9%, 12%, and 18.5%, respectively. This could be attributed to the role of PEG in reducing the agglomeration of MNPs, resulting in smaller particle size and hence more magnetization compared to the pure MNPs sample, where magnetization was minimized due to the decreased surface area because of agglomeration. Santos and his research group observed the same effect of increased magnetization of PEG-GHS-MNPs (67 emu/g) compared to GHS-MNPs (44 emu/g) [29]. It was attributed to the PEG binding that modified the electronic structure of MNPs on the surface, which increased magnetization [29].

### 2.3. Crocetin Loading Characterization

Crocetin was chemically attached to the optimally prepared pegylated MNPs using EDCI-HCl as a crosslinker. Due to the sensitivity of the binding reaction to moisture, both crocetin and EDCI were added over 5, 10, and 20 times more than the theoretically calculated concentration of crocetin needed to bind to the functional groups available on the surface of 12% pegylated MNPs. The amount of available functional groups was calculated based on the amount of polymer loaded onto the surfaces of the MNPs. This amount was estimated from the weight loss results of the optimally prepared pegylated MNPs, using their TGA thermograms. After that, the crocetin-functionalized pegylated MNPs were designated as 5×-Cro-PEG-MNPs, 10×-Cro-PEG-MNPs, and 20×-Cro-PEG-MNPs.

**Figure 5:**
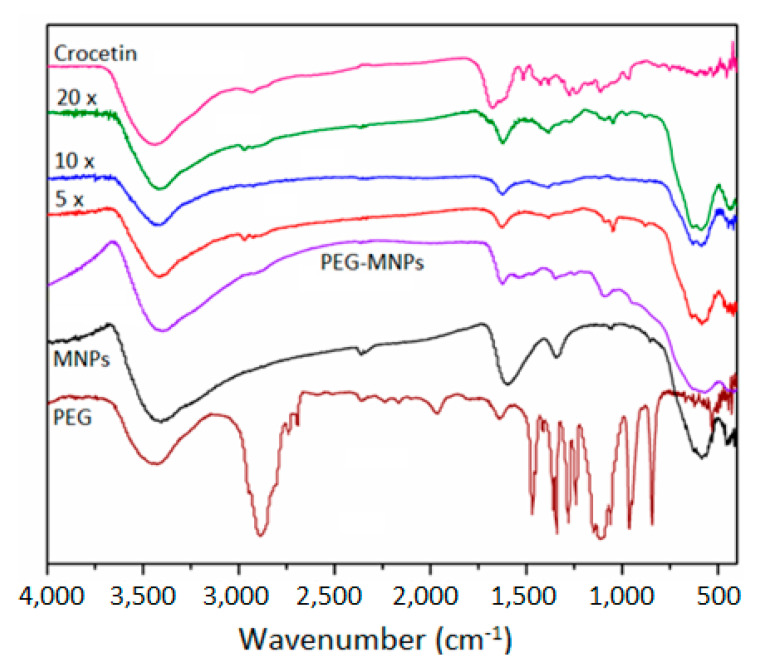
FT-IR spectra of the crocetin-functionalized PEG-coated MNPs as a function of the concentration of crocetin.

The FT-IR spectra of all Cro-functionalized PEG-coated MNPs containing 5×, 10× and 20× of the crocetin coating are presented in Figure 5. A spectrum of pure crocetin was also included in Figure 5 for comparison. Pure crocetin showed several characteristic bands: at 3432 cm^−1^, which is attributed to the hydroxyl group of crocetin; at 2921, 1463, and 1376 cm^−1^, which are characteristic of the -CH group; at 1728 cm^−1^ band, which corresponds to the presence of the carbonyl group (C=O); the 1623 cm^−1^ band, which is distinct from the C=C group; and the 1071 cm^−1^, which is characteristic of the C-O group [35]. Out of these characteristic bands, a band at 1071 cm^−1^, which is attributed to the absorption of the C=C, was observed in the spectra of the crocetin-functionalized PEG-MNPs. Other crocetin-related bands were not observed, which could be attributed to the thinning of the crocetin layer on the coating. Moreover, the three bands at 430, 584, and 622 cm^−1^, which are related to the Fe-O absorption, showed a continuous decrease in intensity with increasing the extent of (PEG + crocetin) coating onto the surfaces of the MNPs.

**Figure 6:**
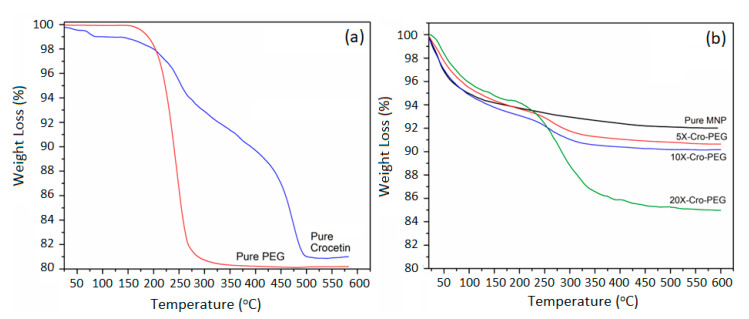
(**a**) TGA thermograms of the crocetin-functionalized pegylated MNPs as a function of the concentration of crocetin, (**b**) TGA thermograms of pure PEG and crocetin.

The TGA thermograms of the Cro-functionalized PEG-coated MNPs are shown in Figure 6, where two major thermal events were observed. The first weight loss event was observed at around 100 °C, corresponding to the loss of physically and chemically absorbed water, while the second thermal event was observed at around 250 °C and is attributed to the thermal decomposition of PEG. In contrast, the TGA thermogram of pure crocetin showed two major thermal events, the first about 200 ± 10 °C and the second around 300 ± 10 °C, as shown in Figure 6a. These events are attributed to the degradation and decomposition of the crocetin molecule, respectively. The weight loss of crocetin overlaps with that of PEG and was thus reflected as a single thermal event in the thermograms of the crocetin-functionalized PEG-coated MNPs. Moreover, since all 5×, 10×, and 20× were prepared from the same batch of 12% PEG-MNPs, hence all pegylated MNPs contain the same amount of PEG. Therefore, the difference in weight loss observed in Figure 6b is attributed to the different loaded amounts of crocetin on the pegylated MNPs. In addition, it was observed that the overall weight loss was increased with the increasing proportion of crocetin initially added. It has been proven, therefore, that the sample containing 20× concentration of crocetin contained the highest amount of crocetin loading, followed by 10×, and lastly 5×.

**Scheme 2:**
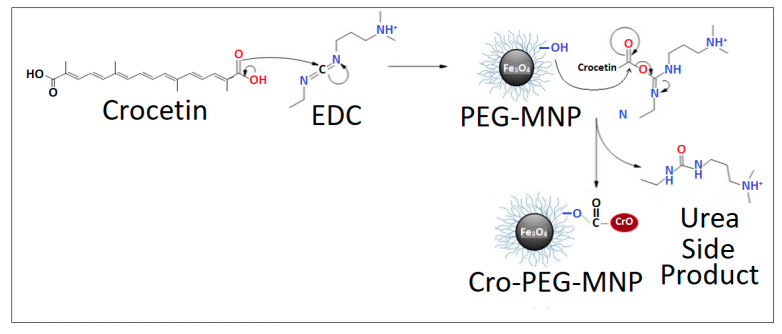
Representation of the possible binding mechanism of crocetin to PEG-MNPs.

The possible binding mechanism of crocetin to PEG is illustrated in Scheme 2. Crocetin initially interacts with EDC.HCl to activate the carboxylic acid group in crocetin. The carbonyl in the carboxylic acid targets the carbodiimide in EDC to form an active O-acylisourea intermediate that is easily displaced by a nucleophilic attack. The hydroxyl group in the PEG serves that purpose and acts as a nucleophile which will then bind to crocetin and give urea as a byproduct. In addition to this mechanism, it is also postulated that crocetin and PEG have the affinity to interact with each other through the formation of H-bonding across their functional groups.

The loading capacity of crocetin was derived from the amount of unbound crocetin in the aliquot of the crocetin-binding procedure. The aliquot was collected and diluted with a known amount of DMSO and then analyzed by a UV-Visible spectrophotometer at 430 nm to estimate the remaining unbound crocetin. A calibration curve of crocetin in DMSO was used to calculate the amount of unbound crocetin. By calculating the difference of the initial amount of crocetin added to PEG-coated MNPs and the amount in the aliquot, we have an estimate of the loading capacity, as presented in Table 1.

### 2.4. Kinetics of Crocetin Release

**Figure 7:**
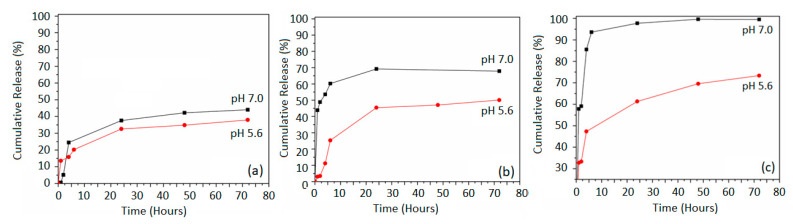
Cumulative release study of crocetin-functionalized pegylated MNPs at different pH values: (**a**) 5×-Cro-PEG-MNP, (**b**) 10×-Cro-PEG-MNPs, (**c**) 20×-Cro-PEG-MNP.

The cumulative release of crocetin from the prepared pegylated MNPs was investigated at pH values of 5.6 and 7.4, and at a physiologic temperature of 37 °C. The lower pH value of 5.6 was selected to simulate the slightly acidic nature of a cancer site, while the pH of 7.4 was chosen to simulate the typical physiologic environment. The cumulative drug release was expressed as the percentage of detached crocetin from the pegylated MNPs as released to the surrounding aqueous media as a function of time. Figure 7 shows the cumulative release (%) of crocetin from the crocetin-functionalized pegylated MNPs at pH 7.4 and pH 5.6 as a function of the initial concentration of crocetin. Figure 7a shows the 5×-Cro-PEG-MNPs cumulative release. A burst effect was observed in the first 6 h, as was previously reported in the literature [29,30]. Within the first 6 h, 25% of crocetin was released at pH 7.4, while 22% of crocetin was released at pH 5.6.

A slower release stage at both pH values followed this stage. At the end of the 3-day testing period, the full release of crocetin for pH 5.6 was 38%, while the total release was 50% at pH 7.4. Figure 7b shows the release of 10× crocetin-functionalized pegylated MNPs. At this concentration, a different trend was observed as a function of the pH of the medium. A burst effect was also observed within the first 6 h for pH 5.6 with a release of 25% of crocetin, while at pH 7.4, 60% was released. This stage was also followed by a slower stage at both pH values. At the end of the 72 h testing period, the total cumulative release for pH 5.6 was 48% and for pH 7.4 was 70%.

Similarly, Figure 7c presents the release of 20× crocetin-functionalized pegylated MNPs. The burst effect reached the maximum after 6 h with pH 7.4; almost 100% of crocetin was released. On the other hand, only 47% of crocetin was released for the same period at pH 5.6. At the end of the testing period, 72% of crocetin was released at pH 5.6.

We observe a higher release of crocetin from the PEG-coated MNPs in the 20× than the 10× and 5× samples. Overall, it is evident that the burst effect is consistent in the first 6 h. The release of crocetin was generally higher at pH 7.4 than pH 5.6. This might be attributed to the increase in cleavage or the hydrolysis of carboxylic esters linkage bonds between crocetin and PEG at pH 7.4 compared to pH 5.6. In acidic conditions, we have a higher concentration of H^+^, which may indicate a weaker probability of hydrophilic attacks on the carboxylic ester group between crocetin and the underlying PEG coating. This shows a lower extent of hydrolysis; hence less crocetin was released.

### 2.5. In Vitro Cytotoxicity

**Figure 8:**
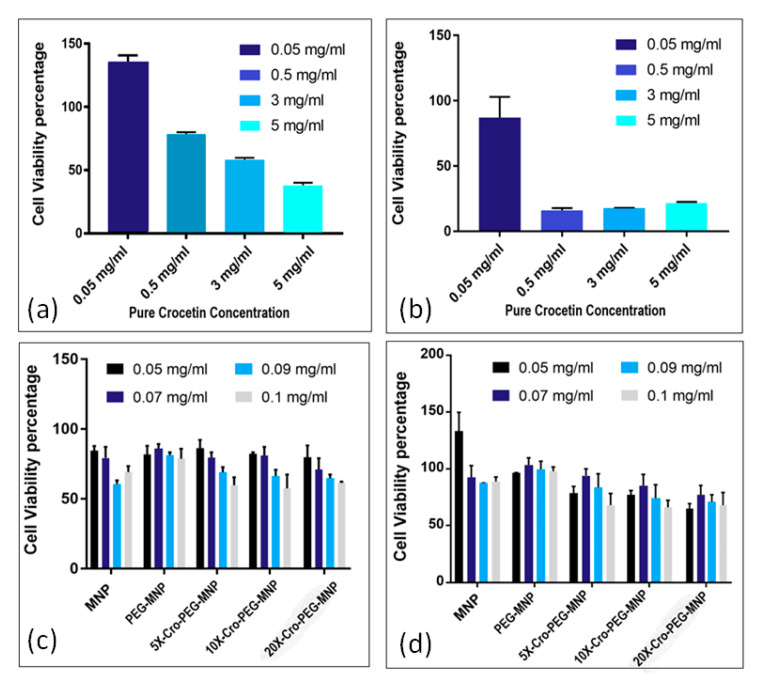
(**a**) Cell viability of HepG2 cells against pure crocetin as a function of the concentration of crocetin after 24 h of culture at 37 °C. (**b**) Cell viability of HepG2 cells against pure crocetin after 72 h of culture at 37 °C. (**c**) Cell viability of HepG2 cells against crocetin-functionalized PEG-coated MNPs after 24 h of culture at 37 °C. (**d**) Cell viability of HepG2 cells against crocetin-functionalized PEG-coated MNPs after 72 h of culture at 37 °C.

In this study, HepG2 cell proliferation and viability were evaluated by MTT assay. Hep2 cells were incubated for 24 and 72 h at four different concentrations of 5×, 10×, and 20× crocetin-functionalized pegylated MNPs, including 0.05, 0.5, 3, and 5 mg/mL as well as free crocetin as the control. The IC50 is calculated to be 0.0817 mg/mL based on the results from 72 h. Figure 8a,b show the MTT assay after 24 and 72 h of HepG2 cells treated with free crocetin. Results show a reduction in the cell viability with increasing the concentration of crocetin. Furthermore, Figure 8c,d show the cell viability results of crocetin-functionalized pegylated MNPs at different concentrations, including 0.05, 0.07, 0.09, and 0.1 mg/mL. Pure MNPs and PEG-MNPs were also tested as control samples. In the 24 h cell viability assay, the as-prepared MNPs exhibited a toxic effect, reducing the cell viability compared to the pegylated MNPs. The reduced toxicity in PEG-MNPs can indicate that coating has limited the nonspecific cell interactions, as compared to the as-prepared MNPs [29]. The same observation was also observed in the 72 h incubation period, reducing the cell viability with IC50 of 0.0634 mg/mL as compared with the pegylated MNPs with IC50 0.1183 mg/mL. In general, the proliferation of HepG2 cells showed a decrease with the increasing dose of each Cro-PEG-MNPs concentration. The calculated IC50s for Cro-PEG-MNPs were 0.1019, 0.0903, and 0.0462 mg/mL of 5×, 10× and 20×, respectively. These findings indicate the potential of the crocetin-functionalized pegylated MNPs for the treatment of hepatic cancer. It is also anticipated that the release of crocetin will be followed by the degradation of the PEG coating and the subsequent degradation of the MNPs into free iron ions that bind to the ferritin protein in the cells. Because of the leaky and pre-mature blood vessels of cancer tissues, the chance of crocetin-functionalized pegylated MNPs accumulating inside these tissues by passive targeting will be higher than in normal tissues. Consequently, this will increase the hydrolysis and release of crocetin to the cancer microenvironment.

## 3. Materials and Methods

### 3.1. Synthesis of Pristine MNPs

Chemicals used to prepare pure MNPs included iron (II) chloride tetrahydrate (FeCl_2_.4H_2_O), iron (III) chloride (FeCl_3_), sodium hydroxide (NaOH), and ammonia solution (NH_4_OH). All reagents were analytical-grade and were purchased from Sigma-Aldrich (St. Louis, MO, USA). Aqueous solutions of 0.3 M ferrous and 0.6 M ferric (1:2 ratio) were separately prepared by dissolving the respective amounts of FeCl_2_.4H_2_O and FeCl_3_ in de-ionized water.

The following revised preparation procedure was carried out [28]. A 5 M aqueous solution of sodium hydroxide was prepared by dissolving the equivalent amount of NaOH in de-ionized water. An amount of 50 ml of aqueous NaOH solution was heated at 60 °C. A mixture containing equal volumes of Fe^2+^ (0.3 M) and Fe^3+^ (0.6 M) was injected into the NaOH solution at a feeding rate of 40 mL/h using a syringe pump with vigorous stirring. A brownish-black precipitate representing the magnetite phase was formed. The whole solution was vigorously stirred for 60 min at a temperature of 60 °C. The ferrofluid was centrifuged at 3000 rpm for 15 min, followed by a successive decantation/washing with 25% ammonia solution three times to maintain high primary conditions. After final decantation, MNPs deposits were collected and dried at 60 °C for 24 h. Dried powders were finely ground to be characterized for their composition, morphology, and thermal and magnetic properties.

### 3.2. Surface Modification of MNPs

Poly (ethylene glycol) (PEG), high purity (>99.9%), with a molecular weight of 3350 Da, obtained from Sigma-Aldrich (USA), was used for the modification of the surfaces of the prepared MNPs. To prepare pegylated MNPs, the latter were first prepared as mentioned in the previous section. After, MNPs were formed and left to stir for 60 min at 60 °C. The product was centrifuged for 15 min and washed thrice with deionized water. MNPs were then re-suspended in 50 mL of deionized water and left under sonication for 90 min. Separately, different amounts of PEG (1 g, 2 g, 3 g, 4 g, 6 g, 8 g, and 12 g) were dissolved in 15 mL DI water. These amounts represent weight percentages of 1.5, 3.0, 4.5, 6.2, 9.0, 12.0, and 18.5%, respectively. The PEG solution was then added to the suspended ferrofluid and left to stir overnight with an overhead mechanical stirrer. The pegylated MNPs were washed three times using a 1:1 mixture of ethanol and DI water. The pegylated MNPs were then dried at 60 °C overnight. The final product was then grounded to a fine powder for further characterization of its composition, morphology, and thermal and magnetic properties.

### 3.3. Functionalization of MNPs with Crocetin

Based on the characterization of the structure, morphology, and thermal and magnetic properties of the pegylated MNPs, optimum coating conditions were selected for further functionalization by crocetin (Fisher Scientific, Vantaa, Finland). The amount of crocetin added was based on the available binding sites on the surface of the polymeric coating. Based on that, 1-ethyl-3-(3-dimethylaminopropyl) carbodiimide hydrochloride (EDC.HCl) (Sigma-Aldrich, USA) was added as a linker, while crocetin was added over 5, 10, and 20 times the available binding sites of PEG. First, EDC was added to crocetin that was dissolved in 1 mL dimethyl sulfoxide (DMSO) (Sigma-Aldrich, USA) and left to stir for 30 min in a dark closed environment. Then, a pre-calculated amount of pegylated MNPs was added and stirred overnight. Then, the crocetin-functionalized pegylated MNPs were centrifuged at 6000× *g* rpm for 15 min. The supernatant was taken out to measure the amount of unbound crocetin. The NPs were washed three times using DI water and centrifuged. The NPs were then re-suspended in DI water, and a representative sample was dried for compositional analysis. At the end of this process, three (3) samples were produced: 5×-Cro-PEG-MNPs, 10×-Cro-PEG-MNPs, and 20×-Cro-PEG-MNPs.

#### Calculation of the Crocetin Loading

Crocetin loading was measured by the indirect method. The amount of unbound crocetin was collected from the aliquot from the reaction tube, and its absorbance was measured at 430 nm using a UV/Visible spectrophotometer. The amount of bound crocetin was measured by the difference between the amount of crocetin added to functionalize the PEG-coated MNPs and the amount of free crocetin in the aliquot after the binding process was complete. Equation (2) shown below was used to calculate the efficiency of crocetin coating:

(2) $$\mathrm{Loading}\;\mathrm{Efficiency}\;\left(\%\right)=\frac{\mathrm{Amount}\;\mathrm{of}\;\mathrm{Loaded}\;\mathrm{Crocetin}}{\mathrm{Amount}\;\mathrm{of}\;\mathrm{Initially}\;\mathrm{Added}\;\mathrm{Crocetin}\;}\times 100$$

### 3.4. Characterization Techniques

Neat and coated MNPs were characterized for their composition, morphology, and properties using various characterization techniques. The composition was studied by X-ray diffraction (XRD), Fourier-transform infrared spectroscopy (FT-IR), and thermogravimetric analysis (TGA). An automated Phillips X-ray diffractometer (Phillips, Amsterdam, Netherland), with a step size of 0.02°, a scan rate of 2° per min, and a scan range from 2θ = 10°–70°, was used on a dry sample. FTIR analysis was conducted using a Nicolet Nexus 470 infrared spectrophotometer (Ramsey, MA, USA), where samples were pre-pressed with KBr, then scanned over the normal range of 4000–400 cm^−1^. Thermogravimetric analysis (TGA) was carried out using a TGA-50 Shimadzu thermogravimetric analyzer (Kyoto, Japan), where pre-weighed powder samples were heated to 600 °C at a heating rate of 20 °C/min using an aluminum pan in air, and the percent weight loss was followed and correlated with the original composition of the NPs. A detailed description of the morphology of the NPs was examined by transmission electron microscopy (TEM; CM10-Phillips Amsterdam, The Netherlands). Samples were prepared for imaging by spreading drops of NPs-diluted dispersion on a copper grid coated with a thin carbon layer and then air-dried. The average particle size of the PEG-MNPs were measured using an ImageJ software as depicted from their respective TEM micrographs. Magnetic properties of the neat and coated NPs were measured as a function of the applied magnetic field at room temperature and were measured using a vibrating sample magnetometer (VSM, Quantum Design Inc., San Diego, CA, USA) instrument.

### 3.5. Kinetic Release Study of Crocetin

The release kinetics of crocetin from the crocetin-functionalized pegylated MNPs were studied in physiological media at pH 5.6 and 7.4. In a typical experiment, 0.1 mg/mL of each sample of 5×, 10×, and 20× crocetin-functionalized pegylated MNPs was dosed in phosphate-buffered saline (PBS) (Sigma Aldrich, USA) at pH 7. Another set of samples was produced with PBS, and pH was adjusted to 5.6 using a 0.1 M HCl solution to mimic the acidic medium of the cancer microenvironment. The solution was then kept in an incubator at 37 °C under constant stirring. At fixed intervals, 1.5 mL was taken out and replaced by fresh PBS with the same pH. Then, the NPs were maintained back at 37 °C after being mixed and re-suspended properly. The concentration of the drug released was determined by UV–Visible spectrophotometry at 430 nm and using a calibration curve of free crocetin [36,37]. The percentage of crocetin released was calculated as in the following Equation (3) [38]:

(3) $$\mathrm{Release}\;\left(\%\right)=\frac{\left(\mathrm{Amount}\;\mathrm{of}\;\mathrm{Free}\;\mathrm{Crocetin}\;\mathrm{After}\;\mathrm{Kinetic}\;\mathrm{Rlease}\right)\;}{\left(\mathrm{Amount}\;\mathrm{of}\;\mathrm{Crocetin}\;\mathrm{Loaded}\;\mathrm{in}\;\mathrm{Polymer}-\mathrm{MNPs}\right)}\times 100$$

### 3.6. In Vitro Cytotoxicity

Hepatocellular carcinoma (HCC) cell line, HepG2, was purchased from American Type Culture Collection (ATCC, Manassas, VA, USA). Cells were maintained in 75 cm^2^ culture flasks using Roswell Park Memorial Institute Medium (RPMI) (Life Technologies, Carlsbad, CA, USA), supplemented with 10% fetal bovine serum (Life Technologies, USA) and 1% penicillin/streptomycin (Life Technologies, Carlsbad, CA, USA). Cells were grown in 5% CO_2_ at 37 °C and 100% humidity.

#### MTT Assay

HepG2 cells were seeded at a density of 6000 cells/well in a 96-well plate and allowed to attach for 24 h. After attachment, the complete growth medium was replaced by a fresh medium with the following treatments. Different concentrations of crocetin, including 0.05, 0.5, 3, and 5 mg/mL, were tested, and additional samples of MNP were tested with concentrations including 0.05, 0.07, 0.09, or 0.1 mg/mL. Incubation was carried out over two time points of 24 h and 72 h. After incubation, 10 μL of MTT (5 mg/mL) was added to each well and then incubated at 37 °C for 3 h. After 3 h, the content was discarded, and 100 μL of dimethyl sulfoxide (DMSO) was added to each well. The 96-well plate was wrapped with foil and placed in the shaker for 15 min at low speed. The vessel was allowed to incubate for 15 min. Afterward, the absorbance was measured at 570 nm. Cell viability was measured using Equation (4) [39]:

(4) $$\mathrm{Cell}\;\mathrm{Viability}\;\left(\%\right)=\frac{\left(\mathrm{Absorbance}\;\mathrm{of}\;\mathrm{Tested}\;\mathrm{Compound}\right)\;}{\left(\mathrm{Absorbance}\;\mathrm{of}\;\mathrm{Control}\right)}\times 100$$

## 4. Conclusions

We investigated the potential of using crocetin-coated pegylated MNPs for the treatment of HCC. The optimization of the PEG coating onto the surfaces of MNPs was carried out and followed using various characterization techniques and via the measurements of the magnetic properties of the coated MNPs. The optimally coated pegylated MNPs were further used as a platform for the immobilization of three different concentrations of crocetin. The release kinetics of crocetin from these newly made formulations were studied under acidic and neutral conditions. Our findings indicated good release kinetics in tumor acidic media based on the in vitro experiments. The new formulations prepared in this study showed a high potential to reduce cell proliferation of the HepG2 liver cancer cell line based on the preliminary in vitro cytotoxicity results. A more detailed investigation of the in vitro and in vivo performance of the crocetin-coated pegylated MNPs is needed.

## Figures and Tables

**Table 1:** Loading capacity of crocetin-functionalized PEG-MNPs via waste analysis.

Cro-PEG-MNP	[Crocetin]_Initial_	[Crocetin]_Loaded_	Loading Capacity (%)
5×-Cro-PEG-MNP	4.9 mg	4.7155 mg	96
10×-Cro-PEG-MNP	9.8 mg	8.57 mg	87
20×-Cro-PEG-MNP	19.6 mg	17.7 mg	90

## Data Availability

All research data related to this article are included in the published work.
